# Supplementary material for: Ammonia-oxidizing bacterial communities are affected by nitrogen fertilization and grass species in native C4 grassland soils
Source: PeerJ. 2021 Dec 16;9:e12592. doi: 10.7717/peerj.12592 (PMC8684740; doi:10.7717/peerj.12592)
Supplement: Supplemental Information 1 — Different lowercase letters indicate significant differences between treatment levels within groups. The letters were only shown in groups that have significant effects. Means were compared using Least Significant Difference (LSD) tests (α = 0.05). [file peerj-09-12592-s001.docx]

**Table S1.** Soil properties in relation to agricultural season, N fertilization rate, and grass species. Different lowercase letters indicate significant differences between treatment levels within groups. The letters were only shown in groups that have significant effects. Means were compared using Least Significant Difference (LSD) tests (α = 0.05).

| Factor^†^ | pH | SWC^‡^  (%) | NH_4_^+^-N | NO_3_^-^-N | DOC | DON | TOC | TN | C: N ratio | N_2_O-N  (g ha^-1^ d^-1^) | NP (μg NO_2_-N gdw^-1^ h^-1^) |
| --- | --- | --- | --- | --- | --- | --- | --- | --- | --- | --- | --- |
|  |  |  | (μg gdw^-1^) | | | | (mg gdw^-1^) | |  |  |  |
| April (G) | 6.27±0.05 | 24.46±0.25^b^ | 10.82±0.49^a^ | 1.51±0.30^a^ | 203.55±4.20^b^ | 18.67±0.56^ab^ | 1.82±0.03 | 21.74±0.43 | 11.94±0.11 | 9.71±0.94^b^ | 0.06±0.01^c^ |
| June (H1) | 6.30±0.06 | 30.16±0.46^a^ | 10.57±0.60^a^ | 2.14±0.46^a^ | 163.85±4.07^c^ | 16.16±0.65^b^ | 1.76±0.06 | 21.18±0.68 | 12.05±0.15 | 24.77±7.44^a^ | 0.11±0.02^b^ |
| August (H2) | 6.18±0.03 | 15.03±0.40^c^ | 7.16±0.35^b^ | 0.83±0.25^b^ | 223.00±5.47^a^ | 22.44±1.98^a^ | 1.84±0.05 | 21.66±0.51 | 11.81±0.12 | 1.64±0.17^b^ | 0.18±0.02^a^ |
| 0N | 6.36±0.05^a^ | 23.27±1.63 | 9.37±0.85 | 0.39±0.16^c^ | 210.18±8.60^a^ | 17.65±1.10 | 1.71±0.04 | 20.73±0.53 | 12.11±0.16 | 7.55±1.49^b^ | 0.06±0.01^c^ |
| 67N | 6.29±0.04^a^ | 23.39±1.66 | 9.53±0.57 | 1.13±0.19^b^ | 196.38±8.02^b^ | 19.07±0.95 | 1.82±0.04 | 21.74±0.49 | 11.94±0.12 | 7.25±1.42^b^ | 0.11±0.02^b^ |
| 202N | 6.12±0.05^b^ | 23.00±1.51 | 9.62±0.53 | 2.77±0.39^a^ | 186.07±5.23^b^ | 20.29±1.83 | 1.87±0.05 | 21.97±0.58 | 11.78±0.09 | 20.56±7.43^a^ | 0.17±0.02^a^ |
| SG | 6.32±0.05^a^ | 23.30±1.31 | 9.52±0.49 | 1.71±0.24 | 195.32±6.71 | 20.28±1.49 | 1.83±0.04 | 21.60±0.41 | 11.79±0.12 | 9.23±1.57 | 0.14±0.02^a^ |
| BB | 6.19±0.03^b^ | 23.14±1.29 | 9.51±0.54 | 1.30±0.33 | 198.11±5.77 | 18.03±0.68 | 1.78±0.04 | 21.46±0.47 | 12.06±0.08 | 14.54±5.11 | 0.1±0.02^b^ |
| 0N-SG | 6.48±0.09 | 22.23±2.72^b^ | 10.03±1.75 | 0.48±0.17 | 201.67±13.80 | 17.34±1.71 | 1.74±0.06 | 21.49±0.81 | 12.36±0.36^a^ | 7.28±1.77 | 0.08±0.02 |
| 67N-SG | 6.35±0.05 | 23.77±2.26^ab^ | 9.38±0.51 | 1.39±0.16 | 194.43±13.99 | 19.97±1.52 | 1.83±0.05 | 21.37±0.54 | 11.67±0.10^b^ | 7.49±2.19 | 0.12±0.02 |
| 202N-SG | 6.19±0.08 | 23.56±2.20^ab^ | 9.32±0.54 | 2.86±0.32 | 191.98±8.07 | 22.55±3.48 | 1.90±0.07 | 21.90±0.84 | 11.53±0.09^b^ | 12.26±3.29 | 0.2±0.03 |
| 0N-BB | 6.27±0.03 | 23.96±2.13^a^ | 8.93±0.87 | 0.34±0.25 | 215.85±11.24 | 17.86±1.51 | 1.70±0.06 | 20.22±0.69 | 11.94±0.12^ab^ | 7.74±2.28 | 0.05±0.01 |
| 67N-BB | 6.23±0.06 | 23.02±2.57^ab^ | 9.69±1.05 | 0.88±0.33 | 198.33±8.76 | 18.18±1.14 | 1.81±0.07 | 22.12±0.82 | 12.21±0.18^ab^ | 7.01±1.94 | 0.09±0.03 |
| 202N-BB | 6.06±0.04 | 22.43±2.20^b^ | 9.92±0.95 | 2.68±0.73 | 180.16±6.50 | 18.04±0.93 | 1.83±0.07 | 22.04±0.85 | 12.02±0.11^ab^ | 28.87±14.38 | 0.15±0.03 |
| Apr-0N | 6.35±0.10 | 24.77±0.52 | 12.29±1.26^a^ | 0.45±0.17 | 220.18±3.73 | 19.97±0.88 | 1.79±0.04 | 21.50±0.48 | 12.01±0.23 | 12.00±1.68^b^ | 0.04±0.01^d^ |
| Apr-67N | 6.31±0.05 | 24.22±0.30 | 10.87±0.51^ab^ | 1.20±0.21 | 204.94±6.82 | 18.24±0.69 | 1.77±0.04 | 21.10±0.71 | 11.95±0.20 | 6.99±0.99^b^ | 0.06±0.01^d^ |
| Apr-202N | 6.16±0.07 | 24.44±0.54 | 9.54±0.38^bc^ | 2.70±0.52 | 188.30±3.30 | 18.00±1.21 | 1.90±0.07 | 22.58±0.89 | 11.87±0.18 | 10.52±1.64^b^ | 0.08±0.01^cd^ |
| Jun-0N | 6.43±0.09 | 29.68±0.74 | 9.36±1.14^bcd^ | 0.67±0.44 | 169.15±6.71 | 15.21±1.12 | 1.60±0.06 | 19.68±1.00 | 12.31±0.43 | 9.25±2.30^b^ | 0.04±0.01^d^ |
| Jun-67N | 6.38±0.09 | 31.13±0.76 | 10.43±1.10^ab^ | 1.59±0.39 | 159.77±9.47 | 16.23±1.30 | 1.75±0.07 | 21.16±1.06 | 12.05±0.15 | 13.09±2.54^b^ | 0.1±0.01^cd^ |
| Jun-202N | 6.12±0.12 | 29.60±0.85 | 11.71±0.84^a^ | 3.90±0.76 | 163.52±4.73 | 16.88±1.04 | 1.90±0.11 | 22.43±1.30 | 11.82±0.16 | 49.38±17.44^a^ | 0.17±0.03^b^ |
| Aug-0N | 6.30±0.04 | 15.35±0.74 | 6.46±0.62^e^ | 0.06±0.04 | 241.21±5.54 | 17.78±2.79 | 1.75±0.10 | 21.01±1.16 | 12.00±0.18 | 1.41±0.43^b^ | 0.11±0.01^bc^ |
| Aug-67N | 6.19±0.06 | 14.83±0.87 | 7.30±0.57^de^ | 0.61±0.27 | 224.43±8.92 | 22.76±1.58 | 1.95±0.08 | 22.96±0.57 | 11.83±0.28 | 1.68±0.36^b^ | 0.17±0.03^b^ |
| Aug-202N | 6.08±0.03 | 14.95±0.53 | 7.61±0.61^cde^ | 1.71±0.47 | 206.38±7.76 | 26.01±4.66 | 1.80±0.06 | 20.89±0.77 | 11.64±0.14 | 1.80±0.12^b^ | 0.27±0.02^a^ |
| Apr-SG | 6.34±0.08 | 24.69±0.36 | 11.30±0.90 | 1.95±0.42^ab^ | 202.78±4.65 | 19.57±0.72 | 1.89±0.05 | 22.14±0.64 | 11.75±0.15 | 8.78±1.17 | 0.08±0.01 |
| Apr-BB | 6.20±0.04 | 24.26±0.36 | 10.39±0.46 | 1.12±0.40^bc^ | 204.24±7.05 | 17.86±0.79 | 1.76±0.03 | 21.38±0.60 | 12.11±0.15 | 10.53±1.45 | 0.04±0.01 |
| Jun-SG | 6.43±0.10 | 30.00±0.73 | 9.69±0.48 | 1.76±0.44^ab^ | 158.05±6.59 | 16.06±0.88 | 1.72±0.07 | 20.55±0.87 | 11.95±0.29 | 17.08±2.46 | 0.13±0.03 |
| Jun-BB | 6.19±0.07 | 30.31±0.63 | 11.35±1.01 | 2.47±0.78^a^ | 169.01±4.65 | 16.24±1.01 | 1.79±0.08 | 21.73±1.03 | 12.13±0.11 | 31.60±13.87 | 0.09±0.02 |
| Aug-SG | 6.20±0.05 | 15.23±0.49 | 7.57±0.57 | 1.42±0.42^b^ | 225.13±6.71 | 25.20±3.81 | 1.89±0.04 | 22.10±0.53 | 11.67±0.17 | 1.82±0.20 | 0.21±0.03 |
| Aug-BB | 6.17±0.04 | 14.84±0.64 | 6.80±0.40 | 0.31±0.15^c^ | 221.09±8.77 | 19.98±1.39 | 1.79±0.08 | 21.26±0.85 | 11.93±0.17 | 1.49±0.28 | 0.16±0.03 |

**Table S1. Continued.**

| Factor^†^ | pH | SWC^‡^  (%) | NH_4_^+^-N | NO_3_^-^-N | DOC | DON | TOC | TN | C: N ratio | N_2_O-N  (g ha^-1^ d^-1^) | NP (μg NO_2_-N gdw^-1^ h^-1^) |
| --- | --- | --- | --- | --- | --- | --- | --- | --- | --- | --- | --- |
|  |  |  | (μg gdw^-1^) | | | | (mg gdw^-1^) | |  |  |  |
| Apr-0N-SG | 6.49±0.20 | 24.06±0.83 | 14.44±2.00 | 0.80±0.14 | 211.52±1.68 | 20.59±0.59 | 1.79±0.07 | 21.43±0.07 | 12.00±0.45 | 9.50±1.58^bc^ | 0.06±0.02 |
| Apr-67N-SG | 6.36±0.08 | 24.22±0.49 | 10.54±0.89 | 1.54±0.24 | 204.90±11.62 | 18.91±1.00 | 1.80±0.01 | 20.96±0.38 | 11.67±0.23 | 5.74±0.82^bc^ | 0.07±0 |
| Apr-202N-SG | 6.23±0.15 | 25.57±0.17 | 9.98±0.61 | 3.13±0.62 | 194.83±2.38 | 19.55±1.75 | 2.04±0.08 | 23.79±1.19 | 11.65±0.13 | 11.34±1.68^bc^ | 0.09±0.02 |
| Apr-0N-BB | 6.25±0.06 | 25.25±0.53 | 10.86±0.84 | 0.21±0.14 | 225.96±1.84 | 19.56±1.49 | 1.79±0.06 | 21.55±0.87 | 12.01±0.26 | 13.66±2.16^bc^ | 0.02±0.01 |
| Apr-67N-BB | 6.26±0.06 | 24.22±0.46 | 11.21±0.62 | 0.87±0.21 | 204.99±9.87 | 17.57±0.97 | 1.73±0.09 | 21.23±1.54 | 12.22±0.28 | 8.23±1.62^bc^ | 0.04±0.01 |
| Apr-202N-BB | 6.09±0.03 | 23.31±0.34 | 9.10±0.37 | 2.27±0.88 | 181.77±2.51 | 16.45±1.36 | 1.77±0.04 | 21.38±1.04 | 12.09±0.31 | 9.69±3.17^bc^ | 0.07±0.02 |
| Jun-0N-SG | 6.58±0.17 | 28.52±0.57 | 9.49±1.55 | 0.52±0.34 | 160.41±4.48 | 15.63±0.08 | 1.58±0.04 | 20.19±2.00 | 12.74±0.94 | 10.01±2.00^bc^ | 0.05±0 |
| Jun-67N-SG | 6.50±0.05 | 31.21±1.14 | 9.51±0.82 | 1.49±0.35 | 150.67±17.47 | 16.25±2.58 | 1.71±0.08 | 20.12±0.86 | 11.80±0.07 | 15.09±3.04^bc^ | 0.12±0 |
| Jun-202N-SG | 6.25±0.21 | 29.76±1.40 | 10.00±0.43 | 2.87±0.69 | 163.85±6.69 | 16.18±0.65 | 1.83±0.17 | 21.23±1.97 | 11.59±0.24 | 23.77±2.08^b^ | 0.2±0.05 |
| Jun-0N-BB | 6.33±0.05 | 30.45±0.96 | 9.28±1.77 | 0.77±0.75 | 174.97±9.88 | 14.93±2.03 | 1.61±0.10 | 19.34±1.08 | 12.03±0.25 | 8.74±3.92^bc^ | 0.04±0.02 |
| Jun-67N-BB | 6.25±0.14 | 31.05±1.25 | 11.35±2.13 | 1.70±0.78 | 168.87±7.78 | 16.21±1.34 | 1.80±0.14 | 22.21±1.94 | 12.31±0.20 | 11.09±4.35^bc^ | 0.08±0 |
| Jun-202N-BB | 5.99±0.11 | 29.44±1.27 | 13.43±0.62 | 4.93±1.16 | 163.18±8.19 | 17.58±2.13 | 1.96±0.16 | 23.64±1.77 | 12.05±0.13 | 74.98±29.33^a^ | 0.14±0.05 |
| Aug-0N-SG | 6.39±0.04 | 14.10±0.16 | 6.15±0.97 | 0.11±0.08 | 233.07±4.94 | 15.80±4.28 | 1.86±0.05 | 22.87±0.40 | 12.33±0.14 | 2.32±0.44^bc^ | 0.13±0 |
| Aug-67N-SG | 6.19±0.05 | 15.87±0.79 | 8.09±0.41 | 1.14±0.27 | 227.73±18.46 | 24.75±1.08 | 1.99±0.06 | 23.02±0.66 | 11.55±0.20 | 1.64±0.35^c^ | 0.18±0.02 |
| Aug-202N-SG | 6.09±0.06 | 15.35±0.96 | 7.99±1.25 | 2.56±0.53 | 217.25±3.50 | 31.92±8.56 | 1.82±0.06 | 20.67±0.68 | 11.36±0.09 | 1.66±0.17^c^ | 0.3±0.02 |
| Aug-0N-BB | 6.24±0.03 | 16.19±0.98 | 6.66±0.87 | 0.02±0.00 | 246.63±7.31 | 19.10±3.83 | 1.68±0.16 | 19.78±1.57 | 11.78±0.18 | 0.80±0.24^c^ | 0.09±0.01 |
| Aug-67N-BB | 6.18±0.12 | 13.79±1.44 | 6.51±0.92 | 0.07±0.05 | 221.13±6.81 | 20.76±2.72 | 1.91±0.16 | 22.91±1.09 | 12.10±0.52 | 1.73±0.71^c^ | 0.16±0.06 |
| Aug-202N-BB | 6.08±0.04 | 14.55±0.58 | 7.22±0.41 | 0.85±0.25 | 195.52±13.07 | 20.10±0.65 | 1.77±0.13 | 21.10±1.56 | 11.92±0.12 | 1.94±0.14^c^ | 0.23±0.03 |

^†^ 0N, no N fertilization; 67N, 67 kg N ha^-1^ fertilization; 202N, 202 kg N ha^-1^ fertilization; SG, switchgrass; BB, big bluestem.

^‡^SWC, soil water content; DOC, dissolved organic C; DON, dissolved organic N; TOC, total organic C; TN, total N; NP, nitrification potential.
