# Supplementary material for: Ammonia-oxidizing bacterial communities are affected by nitrogen fertilization and grass species in native C4 grassland soils
Source: PeerJ. 2021 Dec 16;9:e12592. doi: 10.7717/peerj.12592 (PMC8684740; doi:10.7717/peerj.12592)
Supplement: Supplemental Information 2 — F-values are reported. [file peerj-09-12592-s002.docx]

**Table S2.** Results of mixed model ANOVA (based on GLIMMIX procedure in SAS) testing effects of agricultural season, nitrogen fertilization rate, and grass species on the alpha-diversity of *amoA* genes. F-values are reported.

| Alpha diversity | Chao1 estimator | Observed OTUs | Shannon index | Pielou’s evenness |
| --- | --- | --- | --- | --- |
| Season | **5.21*** | **4.29*** | 0.26 | 0.34 |
| Nitrogen | 1.98 | **17.26***** | **56.65***** | **52.52***** |
| Grass | **9.31**** | **16.87***** | 2.29 | 0.11 |
| Season×Nitrogen | 0.97 | 0.39 | 0.75 | 0.82 |
| Season×Grass | 2.40 | 0.98 | 0.10 | 0.06 |
| Nitrogen×Grass | 1.00 | 1.77 | 1.97 | 2.59 |
| Season×Nitrogen×Grass | 0.32 | 0.71 | 0.57 | 0.53 |

Significance level: * 0.01 < *p*-value ≤ 0.05; ** 0.001 < *p*-value ≤ 0.01; *** *p*-value ≤ 0.001.
