# Supplementary material for: Ammonia-oxidizing bacterial communities are affected by nitrogen fertilization and grass species in native C4 grassland soils
Source: PeerJ. 2021 Dec 16;9:e12592. doi: 10.7717/peerj.12592 (PMC8684740; doi:10.7717/peerj.12592)
Supplement: Supplemental Information 3 [file peerj-09-12592-s003.docx]

**Table S3.** Pearson correlation coefficients among *amoA* genes and transcripts, alpha-diversity of ammonia-oxidizing bacterial community, and soil properties.

|  | pH | SWC^†^ | NH_4_^+^-N | NO_3_^-^-N | DOC | DON | TOC | TN | C: N ratio | N_2_O-N | NP | *amoA* gene | *amoA* transcript |
| --- | --- | --- | --- | --- | --- | --- | --- | --- | --- | --- | --- | --- | --- |
| *amoA* gene | -0.303^*^ | -0.792^**^ | -0.471^**^ | -0.066 | 0.477^**^ | 0.445^**^ | 0.173 | 0.273 | -0.241 | -0.322^*^ | 0.530^**^ | 1 | -0.215 |
| *amoA* transcript | 0.067 | 0.561^**^ | 0.448^**^ | 0.339^*^ | -0.640^**^ | -0.110 | 0.042 | 0.059 | -0.050 | 0.228 | 0.005 | -0.215 | 1 |
| Chao1 estimator | -0.234 | -0.190 | -0.114 | 0.079 | 0.056 | 0.176 | 0.068 | 0.018 | 0.129 | -0.221 | 0.235 | 0.402^**^ | 0.070 |
| Observed OTUs | -0.352^*^ | -0.195 | -0.085 | 0.359^*^ | -0.080 | 0.356^*^ | 0.028 | 0.082 | -0.124 | -0.036 | 0.531^**^ | 0.552^**^ | 0.124 |
| Shannon index | -0.569^**^ | -0.014 | 0.161 | 0.624^**^ | -0.264 | 0.114 | 0.269 | 0.290^*^ | -0.065 | 0.280 | 0.514^**^ | 0.309^*^ | 0.207 |
| Pielou’s evenness | -0.585^**^ | 0.040 | 0.225 | 0.652^**^ | -0.295^*^ | 0.046 | 0.316^*^ | 0.328^*^ | -0.047 | 0.349^*^ | 0.471^**^ | 0.217 | 0.220 |

** Correlation is significant at the 0.01 level (2-tailed).

* Correlation is significant at the 0.05 level (2-tailed).

^†^SWC, soil water content; DOC, dissolved organic C; DON, dissolved organic N; TOC, total organic C; TN, total N; NP, nitrification potential.
