# Supplementary material for: Ammonia-oxidizing bacterial communities are affected by nitrogen fertilization and grass species in native C4 grassland soils
Source: PeerJ. 2021 Dec 16;9:e12592. doi: 10.7717/peerj.12592 (PMC8684740; doi:10.7717/peerj.12592)
Supplement: Supplemental Information 4 [file peerj-09-12592-s004.docx]

**Table S4.** Specific standardized regression weight (path coefficient) for each pathway in the structural equation modeling for ammonia-oxidizing bacteria and soil properties.

| From | To | Path  coefficient | From | To | Path  coefficient | From | To | Path  coefficient |
| --- | --- | --- | --- | --- | --- | --- | --- | --- |
| Season | SWC^†^ | -0.590 | N fertilization | *amoA* gene abundance | 0.223 | DON | TN | 0.115 |
| N fertilization | AOB community | -0.679 | TN | DOC | 0.315 | SWC | DOC | -0.776 |
| AOB community | Observed OTUs | -0.615 | DON | DOC | 0.184 | SWC | *amoA* gene abundance | -0.711 |
| N fertilization | Nitrate | 0.317 | TOC | *amoA* gene abundance | 0.186 | Season | DOC | -0.339 |
| Season | ammonium | -0.348 | AOB community | *Nitrosospira* sp. 56-18 | -0.886 | N fertilization | DOC | -0.416 |
| SWC | ammonium | 0.354 | AOB community | *Nitrosospira* sp. APG3 | -0.177 | Observed OTUs | *amoA* gene abundance | 0.604 |
| SWC | Nitrate | 0.427 | AOB community | *Nitrosospira multiformis* | -0.842 | Shannon index | *amoA* gene abundance | -0.394 |
| AOB community | Nitrate | -0.474 | AOB community | *Nitrosospira* sp. Nsp14 | 0.882 | *amoA* gene abundance | *amoA* transcript abundance | 0.541 |
| Grass | Observed OTUs | -0.329 | SWC | N_2_O emission | 0.723 | Ammonium | *amoA* transcript abundance | 0.331 |
| Grass | Nitrate | -0.193 | Grass | pH | -0.394 | Nitrate | Chao1 index | -0.366 |
| SWC | DON | -0.552 | Observed OTUs | Chao1 index | 0.978 | Season | Chao1 index | -0.266 |
| Observed OTUs | Evenness | 0.297 | AOB community | N_2_O emission | -0.294 | DON | pH | -0.329 |
| Ammonium | Evenness | 0.268 | SWC | *amoA* transcript abundance | 0.433 | TOC | pH | -0.239 |
| Nitrate | DON | 0.366 | DOC | *amoA* transcript abundance | -0.493 | Evenness | Shannon index | 0.836 |
| AOB community | Evenness | -0.690 | AOB community | Nitrification potential | -0.386 | Nitrate | TN | 0.132 |
| AOB community | Shannon index | 0.049 | Season | Nitrification potential | 0.599 | Grass | Nitrification potential | -0.267 |
| Observed OTUs | Shannon index | 0.263 | N fertilization | Nitrification potential | 0.204 | Ammonium | N_2_O emission | 0.167 |

^†^SWC, soil water content; DOC, dissolved organic C; DON, dissolved organic N; TOC, total organic C; TN, total N; AOB, ammonia-oxidizing bacteria.
