# Supplementary material for: Ammonia-oxidizing bacterial communities are affected by nitrogen fertilization and grass species in native C4 grassland soils
Source: PeerJ. 2021 Dec 16;9:e12592. doi: 10.7717/peerj.12592 (PMC8684740; doi:10.7717/peerj.12592)
Supplement: Supplemental Information 5 [file peerj-09-12592-s005.docx]

**Table S5.** Standardized total effects from the structural equation model relating agricultural season, grass species, N fertilization rate, and soil physicochemical properties on ammonia-oxidizing bacterial abundance, activity, diversity, and major community composition, as well as nitrification potential and N_2_O emission.

| **Factors** | **Grass**  **species** | **Nitrogen**  **fertilization** | **Season** | **SWC^†^** | **Nitrate** | **Ammonium** | **DON** | **TOC** | **TN** | **DOC** |
| --- | --- | --- | --- | --- | --- | --- | --- | --- | --- | --- |
| **Observed *amoA* OTUs** | -0.329 | 0.417 | 0.000 | 0.000 | 0.000 | 0.000 | 0.000 | 0.000 | 0.000 | 0.000 |
| **Evenness** | -0.098 | 0.592 | -0.149 | 0.095 | 0.000 | 0.268 | 0.000 | 0.000 | 0.000 | 0.000 |
| **Shannon index** | -0.168 | 0.572 | -0.125 | 0.079 | 0.000 | 0.224 | 0.000 | 0.000 | 0.000 | 0.000 |
| **Chao1 index** | -0.251 | 0.174 | -0.173 | -0.157 | -0.366 | 0.000 | 0.000 | 0.000 | 0.000 | 0.000 |
| ***amoA g*ene abundance** | -0.132 | 0.250 | 0.469 | -0.742 | 0.000 | -0.088 | 0.000 | 0.186 | 0.000 | 0.000 |
| ***amoA* transcript abundance** | -0.060 | 0.302 | -0.265 | 0.565 | -0.060 | 0.283 | -0.108 | 0.101 | -0.155 | -0.493 |
| **N_2_O emission** | 0.000 | 0.199 | -0.520 | 0.782 | 0.000 | 0.167 | 0.000 | 0.000 | 0.000 | 0.000 |
| **Nitrification potential** | -0.267 | 0.466 | 0.599 | 0.000 | 0.000 | 0.000 | 0.000 | 0.000 | 0.000 | 0.000 |
| **AOB community** | 0.000 | -0.679 | 0.000 | 0.000 | 0.000 | 0.000 | 0.000 | 0.000 | 0.000 | 0.000 |
| ***Nitrosospira* sp. Nsp14** | 0.000 | -0.598 | 0.000 | 0.000 | 0.000 | 0.000 | 0.000 | 0.000 | 0.000 | 0.000 |
| ***Nitrosospira multiformis*** | 0.000 | 0.572 | 0.000 | 0.000 | 0.000 | 0.000 | 0.000 | 0.000 | 0.000 | 0.000 |
| ***Nitrosospira* sp. APG3** | 0.000 | 0.120 | 0.000 | 0.000 | 0.000 | 0.000 | 0.000 | 0.000 | 0.000 | 0.000 |
| ***Nitrosospira* sp. 56-18** | 0.000 | 0.601 | 0.000 | 0.000 | 0.000 | 0.000 | 0.000 | 0.000 | 0.000 | 0.000 |

^†^SWC, soil water content; DON, dissolved organic N; TOC, total organic C; TN, total N; DOC, dissolved organic C; AOB, ammonia-oxidizing bacteria.
